# Book Reviews

**Published:** 1831-10

**Authors:** 


### CRITICAL ANALYSES.

Quæ laudanda forent, et quæ culpanda, vicissim

Illa, prius, cretâ ; mox hæc, carbone, *notamus*.—PERSIUS.

### *Pathological and Practical Researches on Uterine Inflammation in Puerperal Women.* By Robert Lee, M.D. F.R.S. &c. (*Med. Chir. Trans.* vol. xvi. part ii.)

It would be a waste of time to direct the attention of our readers, by any formal preliminary observations, to the conflicting opinions that have been maintained by physicians as to the real nature of the disease termed "puerperal fever." Modern investigations have thrown a new light on the subject, and have satisfactorily shewn how imperfect were the notions which were previously entertained of the pathology of this disease. In France especially, inquiries upon this important topic have been diligently and ably conducted;* and in this country the distinguished author of the paper we are about to analyze has very materially contributed to enlarge our knowledge of the real nature of the disease, and to explain the cause upon which its most formidable symptoms are dependent.

In October 1829, Dr. Lee laid before the Medico-Chirurgical Society a very valuable essay on Inflammation of the Veins of the Uterus. He inferred, from the appearances he had witnessed in numerous dissections, that uterine phlebitis is of far more frequent occurrence than had before been suspected, and that to it must be referred many of the fatal disorders of puerperal women, which have usually been comprehended under the vague designation of puerperal fever, or peritonitis. At that period he had also arrived at the following conclusion, which his subsequent experience has fully confirmed: "that inflammation of the uterus, and its appendages, must be considered as essentially the cause

* Vide the valuable series of papers by M. Tonnelle on "Puerperal Fevers which occurred at La Maternité," in our last volume.

of all the destructive febrile affections which follow parturition, and that the various forms they assume, inflammatory, congestive, or typhoid, will, in a great measure, be found to depend on the serous, muscular, or venous tissue of the organ having become affected."†

From the 1st of January, 1827, to the 1st of March, 1831, 112 cases of well-marked uterine inflammation have fallen under his observation in the British Lying-in Hospital, and in public and private practice in the western districts of this metropolis. The symptoms and progress of these cases were watched with the closest attention; the effects of remedies were observed; and, where death took place, the alterations of structure which existed in the uterine and other organs were carefully examined.

"Of forty cases which have proved fatal, the bodies of thirty-four have been examined, and in all of these, which had presented during life the characteristic symptoms of what has been usually denominated Puerperal Fever, there existed some morbid change from inflammation either in the peritoneal coat of the uterus, or of the uterine appendages, in the muscular tissue, the veins or absorbents of the uterus, to account in a complete and most satisfactory manner for all the constitutional disturbance which had been observed. The peritoneum and uterine appendages were found inflamed in twenty-six cases, in fourteen there existed uterine phlebitis, in eight inflammation and softening of the muscular tissue of the organ, and in four the absorbents were distended with pus. The results of these observations, as far as they go, are therefore decidedly opposed to the opinion now generally prevalent in this country, that there is a specific fever, which attacks puerperal women, and which may arise independent of any local affection in the uterine organs, and frequently prove fatal, without leaving any perceptible change in the organization of their different textures." (P. 379.)

The principal modifications of inflammation of the uterus in puerperal women, which Dr. Lee has observed, are, 1st, inflammation of the peritoneal covering of the uterus, and of the general peritoneal sac; 2dly, inflammation of the uterine appendages, ovaria, Fallopian tubes, and broad ligaments; 3dly, inflammation of the muscular or proper tissue of the uterus; 4thly, inflammation and suppuration of the veins and absorbent vessels of these organs. These varieties of uterine inflammation may occur wholly independent of each other, though they are most frequently met with in combination. Peritonitis seldom occurs without some degree of inflammation of the uterine appendages; but both these

* Medico-Chir. Trans. vol. xv. page 465.

textures may be severely affected, when the muscular coat of the uterus and the veins are wholly exempt from disease. The venous and muscular tissues of the uterus are also liable to severe attacks of inflammation, without any corresponding affection of the peritoneum by which they are covered; though it most frequently happens that inflammation, when set up, either in the veins or muscular coat, involves also the peritoneum.

*Inflammation of the peritoneal Covering of the Uterus, and of the general peritoneal Sac.* The effects produced by inflammation of the peritoneal coat of the uterus in puerperal women, do not essentially differ from those produced by ordinary peritonitis in the male sex.

"Inflammation of the peritoneal coat of the uterus is characterized by great tenderness of the surface of the organ, increased on pressure, and by pyrexia more or less severe. In every instance, on a careful examination of the uterine region, there has been more or less pain in it, increased by pressure, with constitutional disturbance; though it must be admitted that the pain and febrile symptoms have varied greatly in intensity.

"When the attack of peritonitis is severe, the patient commonly lies upon the back, with the knees drawn up to the trunk of the body. At the onset of the disease, the abdomen is generally soft and flaccid, and, except in the region of the uterus, not affected by pressure. Dr. Hulme has described the pain as affecting the whole hypogastric region from the commencement of the attack, but this is the case only where the disease has made considerable progress, or has extended from the uterus to the general investing membrane of the abdomen. Though an enlarged and painful state of the uterus be never altogether wanting, yet the pain often undergoes exacerbations similar to after pains, and is often mistaken for them by careless observers, and the disease is thus overlooked till a great part of the peritoneal sac is inflamed, and the case in consequence is rendered hopeless.

"The whole abdomen at length becomes distended, tympanitic, and occasionally exquisitely painful on pressure; vomiting of dark green coloured fluid substances follows; the pulse grows rapid and feeble, the tongue dry and brown, the lips and teeth covered with dark sordes, diarrhœa frequently supervenes, and death ensues at no very remote period.

"The invasion of pain in the uterus is sometimes sudden, at other times the ordinary increased sensibility of the uterus, subsequent to the efforts of natural labour, or after pains, passes slowly and insensibly into the acute pain increased by pressure, which is the great characteristic symptom of uterine inflammation. Most frequently the accession of the disease is marked by rigors, partial or general, sometimes so slight as scarcely to be perceived by the patient, at other times so violent as to produce strong succussions of the whole body. The cold shivering after a longer or shorter duration passes away, and is succeeded by great heat of the surface, acceleration of the pulse and of the respiration, thirst, sometimes nausea, and vomiting, and intense pain across the forehead. The rigors precede, accompany, or follow the increased sensibility of the uterus. In some of the most severe cases, there has been no distinct rigor; but a quick pulse, hot skin, and hurried respiration, have rapidly succeeded to the uterine pain. In some of the most unfavorable cases, the extremities have been cold, and the countenance anxious and pallid, after the disease has been completely formed." (P. 383.)

There is no uniformity in the state of the tongue in puerperal peritonitis. The lochia are sometimes completely suppressed, and sometimes only diminished in quantity. The mammæ usually become flaccid; "yet, in some fatal cases, the milk has been secreted till a short period before death." Dr. Lee admits, that in some cases it is difficult to distinguish puerperal peritonitis from the irregular contractions of the uterus, which constitute after pains and hysteralgia: but he believes that, where the pulse is accelerated, the remissions of pain incomplete, the lochia scanty or suppressed, in a large proportion of cases we shall arrive at a correct diagnosis, by considering the peritoneal coat of the uterus, or its deeper-seated tissues, in a state of congestion or inflammation.

"Intestinal irritation, depending on a disordered state of the bowels, is also liable to be mistaken for peritonitis, and treated by bloodletting to the injury of the patient. In this affection the abdominal pain is diffused, it is rather a griping than acute pain: it does not commence in the region of the uterus, nor is it aggravated by pressure. The abdomen is generally soft, puffy and distended; the tongue is loaded: there is thirst and headach, the lochia and milk are not suppressed, the febrile attack is usually preceded by evident signs of great intestinal derangement, flatulence, nausea, vomiting, constipation or diarrhœa. The constitutional disturbance attending intestinal irritation comes on about the end of the first week, whereas peritonitis manifests itself most frequently before the fourth day subsequent to delivery. The reaction which succeeds to uterine hemorrhage cannot easily be confounded with puerperal peritonitis. The morbid sensibility of the uterus, which characterizes inflammation, and the other symptoms already described, are here entirely wanting." (P. 386.)

*Inflammation of the uterine Appendages, Ovaria, Fallopian Tubes, and broad Ligaments.* In one case only has Dr. Lee found the uterine appendages free from disease, where the peritoneal covering of the uterus has been inflamed, but frequently the peritoneum has been slightly affected, where the appendages of the uterus have been extensively disorganized. The surface of the broad ligaments, ovaria, and Fallopian tubes, has been red and vascular, and partially or completely imbedded in lymph or pus. Between the folds of the broad ligaments, effusion of serous or purulent fluids have also been found. Various important changes were seen in the ovaria.

"The ovarium appeared in one instance which I observed to be converted into a large purulent cyst, which had contracted adhesions with the abdominal parietes, and discharged its contents exteriorly through an ulcerated opening. In another case which proved fatal, the inflamed uterine appendages, agglutinated together by lymph, had contracted adhesions with the peritoneum at the brim of the pelvis, the inflammation had extended to the cellular membrane, exterior to the peritoneum, and had given rise to an extensive purulent deposit in the course of the psoas and iliacus internus muscles, as in lumbar abscess.

"In two other individuals, who ultimately recovered, the purulent matter, formed in the situation of the psoas and iliacus internus muscles from inflammation of the uterine appendages, made its way through an opening at the upper part of the thigh. Contraction of the thigh on the trunk took place in both these cases, and continued for several months, but disappeared on the recovery of the patient. The uterus remains immoveably fixed to the right side of the pelvis, in a woman who, six months ago, had a severe attack of inflammation of the peritoneum, and uterine appendages of the same side, a few days after delivery." (P. 388.)

To illustrate the morbid changes which occur in the peritoneal coat of the uterus, and in the uterine appendages of puerperal women, Dr. Lee relates nine fatal cases, the two first of which we give as examples.

"I. Mrs. Groom, æt. twenty-eight, No. 13, Little Coram street, was delivered of her first child on the 6th March, 1827. On the 8th, great tenderness of the uterine region took place, with suppression of the lochia and febrile symptoms, which, being supposed by her medical attendant to depend on spasmodic contractions of the uterus, were treated with anodynes, and warm fomentations to the hypogastrium.

"On the 10th, (the fourth day after her confinement, and the first on which I saw her,) the abdomen was tympanitic and exquisitely painful on pressure. The pulse 140, and feeble, the extremities cold, countenance haggard. There was incessant vomiting of a dark green fluid, with diarrhœa, and she died in the afternoon.

"*Dissection.* The stomach and small intestines were inflated with gas. The peritoneum covering the fundus and posterior part of the uterus, was of a bright red colour, and the cellular membrane underneath it in this latter situation was infiltrated with pus. The peritoneal coat of the small intestines was highly vascular in different parts, and the surface of the liver was partially covered with lymph. The uterine appendages on both sides were covered with pus and lymph, and the lumbar region contained about a pint of a wheyish coloured turbid fluid. The consistence of the spleen was remarkably soft.

"CASE II. Elizabeth Marshall, æt. twenty-three, No. 3, Crown place, Soho; was attacked on the 4th of March, 1827, (the third day after her delivery,) with rigors, headach, vertigo, and sense of exquisite tenderness in the hypogastrium and right groin. The milk and lochia soon disappeared; bloodletting was employed on the 8th, and leeches were applied to the region of the uterus, but the tenderness gradually extended over the whole abdomen, which became as large as before delivery, and tympanitic. The pulse was rapid and intermitting. The tongue covered with a brown fur, singultus, and vomiting of dark coloured matter succeeded, and she died on the twelfth day after the attack.

"*Dissection.* The uterus with its appendages, and the small intestines, were all imbedded in thick masses of lymph, and closely adhered to one another. The omentum, colon, and peritoneum lining the abdominal muscles, were vascular, of a deep red colour, and partially coated with false membranes. About ℥x. of sero-purulent fluid were contained in the cavity of the abdomen. The deeper seated tissues of the uterus were healthy." (P. 390.)

*Inflammation and Softening of the proper or muscular Tissue of the Uterus.* The descriptions of these morbid changes do not materially differ from the account given by M. Tonnellé.* Dr. Lee, however, says, "that the destruction of the healthy organization of the proper tissue of the uterus, in puerperal women, is the consequence of an inflammatory process, may be inferred from the symptoms which accompany the disease, and from its occurring in combination with the other varieties of uterine inflammation." M. Tonnellé speaks with but little confidence upon this subject, but he is inclined to think "that inflammation in such cases is but an accessory phenomenon, and a sort of veil, behind which is concealed the really active cause. It is difficult to determine the precise nature of this cause: we have many reasons for believing that it depends upon a vitiated condition of the blood, but we confess that facts are yet wanting to support this, at present, hypothetical opinion."† Inflammation of the muscular coat of the uterus most frequently commences with pain in the hypogastrium, irregularity of the lochial discharge, rigors, and other symptoms of fever.

"The countenance becomes pallid, and is usually expressive of great anxiety and distress. There is often severe headach, with delirium and other affections of the brain and nervous system, and

* London Med. and Phys. Journal, for October 1830, p. 300. † Ibid.

so violent have these been in some cases, that the local affection of the uterus has completely escaped detection during life. The skin is hot and dry, and sometimes of a peculiar sallow tinge, the pulse is rapid and feeble. The respiration hurried, with remarkable prostration of strength. The tongue soon becomes foul. The lips covered with sordes: occasional vomiting is experienced. The progress of the disease in some cases is rapid, in others it runs its course more slowly, being protracted to the eighth or tenth day.

"It must be admitted, that the diagnosis of this variety of uterine inflammation, particularly where it is complicated with peritonitis or phlebitis, which is frequently the case, is difficult or even impossible. If the attack of inflammation of the muscular coat be sudden and violent, it becomes so speedily complicated with peritonitis more or less acute, that the symptoms are readily confounded together, and it is impossible to distinguish with certainty the symptoms which are to be referred to peritonitis, and those which result from the affection of the muscular coat. The prostration of strength, the alteration of the features, which often exist from the commencement, the feebleness and rapidity of the pulse, the irregular fœtid state of the lochia, are not such constant symptoms as to be pathognomonic, and may arise from other causes. Hence it will appear that the most attentive consideration of the phenomena will not lead us to any certain conclusion as to the nature of the affection, and as in many other diseases, we can only determine its precise character by the history of its origin and progress, and by the alterations of structure discovered after death. In all the cases of this affection which I have observed, the resources of nature and of art have proved equally unavailing in arresting its fatal course. The active inflammatory symptoms, which commonly manifest themselves at the commencement of the attack, pass speedily away, whatever plan of treatment be adopted, and are rapidly succeeded by symptoms of exhaustion. Where the disease is not complicated with inflammation of the peritoneum, the symptoms are not such as to indicate the necessity for the employment of venesection; and in one case where it was adopted freely, the abstraction of the blood was followed by speedy death. In other cases, where the opposite plan of treatment was had recourse to, the fatal result seemed to be less speedy, though equally certain." (P. 404.)

Dr. Lee mentions some cases, from which it appears that softening of the uterine parietes may occasionally take place during utero-gestation, as well as subsequent to delivery.

*Inflammation of the Veins and Absorbents of the Uterus.* The absorbent vessels of the uterus, and receptaculum chyli, were observed, by Mr. Cæsar Hawkins, to be filled with fluid pus, in a case of fatal uterine inflammation subsequent to delivery, which occurred in St. George's Hospital, in July 1829. Since that period, Dr. Lee has found the absorbents in the vicinity of the uterus distended with pus in four cases, and in three of these there existed inflammation and suppuration of the veins.

The late valuable researches of M. Tonnellé* and others have proved that inflammation of the absorbents of the uterus, of the receptaculum chyli, and thoracic duct, occurs not unfrequently in puerperal women, and gives rise to the same constitutional disturbance as uterine phlebitis.

"The presence of purulent fluid in the veins of the uterus after parturition, was pointed out many years ago by Meckel, Schwilgué, Wilson, and J. Clarke; but none of these authors appear to have been aware of the important fact, which has recently been demonstrated by numerous observations, that a large proportion of the cases usually termed low childbed fever, or typhoid puerperal fever, arise from inflammation and suppuration of the uterine veins. Exclusive of the cases which have been recorded in the 15th volume of the Transactions of this Society, ten fatal examples of this insidious and most dangerous affection have fallen under my notice since November 1829; and from an examination of all these cases, it appears that the symptoms of uterine phlebitis correspond in a striking manner with the symptoms assigned by the earlier writers to the putrid puerperal fever, or malignant forms of typhus after delivery." (P. 411.)

Tonnellé states, that in 1829, during the prevalence of the fatal epidemic in the Maternité, inflammation of the veins and lymphatics of the uterus occurred in 132 out of 222 cases, which were examined after death; and that, in 197 cases of the whole, some important alteration of structure was discovered in the uterine organs.† In further confirmation of these facts, Dr. Lee refers to M. Duplay, who "met with eighteen cases of inflamed lymphatics, with or without inflammation of the veins; and in all of these the constitutional phenomena were those which characterise phlebitis in other organs of the body, and in the other sex.

"In women who have enjoyed good health during pregnancy, and in whom the process of parturition has been easily accomplished, uterine phlebitis occasionally commences within twenty-four hours after delivery, with pain more or less acute in the region of the uterus, accompanied or followed by a severe rigor, or a succession of rigors, suppression of the lochial discharge, acceleration of the pulse, cephalalgia, or slight incoherence of ideas, with an insuperable sensation of general uneasiness, and sometimes by nausea and vomiting. These symptoms, after a short duration, are succeeded by increased heat of the body, tremors of the face

* London Med. and Phys. Journal, July 1830, p. 10.

† Ibid. October 1830, p. 302.

and limbs, rapid feeble pulse, anxious and hurried respiration, great thirst, with brown dry tongue, and frequent vomiting of green-coloured matters. The sensorial functions usually become much affected, and there is a state of drowsy stupor or violent delirium and agitation, which terminates in exhaustion. The whole surface of the body not unfrequently assumes a peculiar sallow or deep yellow colour, the abdomen becomes swollen and tympanitic, and some of the remote organs of the body, the brain, heart, lungs, liver, and spleen, or the articulations and cellular membrane of the extremities suffer disorganization, from a rapid and destructive congestion, inflammation, or gangrene.

"At other times, inflammation of the uterine veins commences at a later period after delivery than above mentioned, and in a much more obscure and insidious form, without either pain or sense of uneasiness in the region of the uterus, or any other local symptom by which the affection can be recognized. The uterus may return to its usual reduced volume after delivery, the lochial discharge may continue to flow, and the inflammation and suppuration of the veins, which have caused the whole of the violent constitutional disturbance and destructive lesions in distant parts of the body, may be wholly overlooked during life. In several cases which I shall now relate this occurred, and wine, opium, brandy, and sulphate of quinine, with other stimulants, were liberally administered by the medical attendants, to obviate the debility supposed to be caused by a specific fever, without any local affection of the uterine organs.

"Inflammation of veins rarely takes place in any part of the body where it cannot be referred to a wound, or to a specific cause, externally applied to the coats of the vessels. In uterine phlebitis the inflammation cannot, it is true, invariably be traced to the orifices of the veins where the placenta adhered to the inner surface of the uterus, yet it scarcely admits of a doubt but that the frequent occurrence of the disease is the effect of the communication indirectly established between the venous system and the atmospheric air from the separation of the placenta after delivery. In consequence of this separation, the uterine veins are placed in a condition analogous to that of the great veins of the extremities after amputation and extensive wounds, which condition experience has proved to be favorable to the production of inflammation; and inflammation, being once excited in the vessels, may extend along the continuous membrane of the uterine veins to the spermatic or hypogastric veins, and from thence to the vena cava, and its principal branches returning the blood from the lower extremities." (P. 414.)

This attempt to account for the occurrence of uterine phlebitis after delivery is original and ingenious, but we think not altogether satisfactory. If uterine phlebitis arise in the manner supposed by Dr. Lee, it is extraordinary that it does not occur much more frequently than it really does; for in *every* case after delivery that condition of the parts must exist, which he presumes sufficient to give origin to the disease.

"'The veins which return the blood from the uterus and its appendages,' as I formerly remarked, 'may be either wholly or in part inflamed; generally however, and this is a circumstance in the history of uterine phlebitis deserving particular attention, the inflammation attacks the spermatic veins alone, and for the most part, the one only on that side of the uterus to which the placenta has been attached;* and it may either confine itself to a small portion of the vessel, or extend throughout its whole course from the uterus to the vena cava. The usual consequences of inflammation of veins are then apparent, viz. injection and condensation of the cellular membrane in which they are imbedded, thickening, induration, and contraction of their coats, and the deposition of lymph mixed with pus and coagula of blood within their cavities.

"'The same is the case with regard to the hypogastric veins, one only being generally affected. These veins are, however, rarely inflamed in comparison with the spermatic, and this would seem to depend on the latter veins being invariably connected with the placenta, to whatever part of the uterus it may happen to be attached.' From these facts we have an explanation of the local and constitutional phenomena of phlegmasia dolens, which invariably arises from an extension of the inflammation from the hypogastric to the iliac and femoral veins. (P. 417.)

Nine cases of uterine phlebitis are detailed by Dr. Lee, with the appearances on dissection. In some of these instances, the absorbent vessels of the uterus were also inflamed.

We now come to the consideration of another part of the subject, the *causes* of uterine inflammation, which are generally involved in great obscurity. It most frequently arises where none of the ordinary causes exist, as injuries inflicted on the uterus by severe, protracted, and instrumental labour; the forcible introduction of the hand into the uterus; exposure to cold, &c.; and we are compelled to refer it to some peculiar constitution of the atmosphere, or to contagious miasmata. With respect to contagion as a cause of the disease, the utmost diversity of opinion has existed.

* M. Dance makes the same observation; Archives, Fevrier 1829, p. 189. "Tres souvent cette phlébite est, pour ainsi dire, uni-latérale."—"Cette particularité nous a paru dépendre des variations que subit le placenta relativement a son point d'insertion; plus rapproché d'un coté de la matrice que de l'autre, il laisse, après son decollement, des veines à decouvert qui, venant a s'enflammer communiquent plus directement avec les veines de ce cotè.

Editor.

"It is difficult to reconcile this contradictory evidence, and the facts I have myself observed, though they have inclined me to adopt the opinion that the disease is sometimes communicable by contagion, yet they have not been sufficiently numerous, and of so decisive a character, as to dispel every doubt on the subject. In many cases it has occurred in the most destructive form, where the idea of contagion could not be entertained.

"In the last two weeks of September 1827, five fatal cases of uterine inflammation came under my observation. All the individuals so attacked had been attended in labour by the same midwife, and no example of a febrile or inflammatory complaint of a serious nature occurred during that period among the other patients of the Westminster General Dispensary, who had been attended by the other midwives belonging to the institution.

"On the 16th of March, 1831, a medical practitioner, who resides in a populous parish in the outskirts of London, examined the body of a woman who had died a few days after delivery, from inflammation of the peritoneal coat of the uterus. On the morning of the 17th of March, he was called to attend a private patient in labour, who was safely delivered the same day. On the 19th, she was attacked with the worst symptoms of uterine phlebitis: severe rigors, great disturbance of the cerebral functions, rapid feeble pulse, with acute pain of the hypogastrium, and peculiar sallow colour of the whole surface of the body. She died on the fourth day after the attack, the 22d of March; and between this period and the 6th of April, Mr.—— attended two other patients, both of whom were attacked with the same disease in a malignant form, and speedily fell victims to it." (P. 438.)

Dr. Lee very properly observes, that, whatever conclusion we may arrive at on the contagious or non-contagious nature of the disease commonly termed puerperal fever, it cannot affect the view he has taken of its proximate cause, or essential nature; for the symptoms, morbid appearances, and effects of remedies, all prove, whatever the nature of the remote cause may be, that it acts by exciting inflammation of the uterine organs.

"With regard to the nature of this inflammation, it is difficult to determine whether it be of a common or specific kind. It certainly arises where individuals are not exposed to the ordinary causes of inflammation, and it often reigns as an epidemic, particularly in hospitals; and in this respect it resembles hospital gangrene, erysipelas, and other specific inflammatory diseases, which are generally supposed to depend on a vitiated state of the atmosphere. Like these diseases, too, it ceases, without any assignable cause, perhaps for several years, and then reappears in the same establishment, and is attended with the same destructive consequences." (P. 441.)

At the close of the paper, Dr. Lee gives an abstract of the history of 112 cases of uterine inflammation; from whence it appears, that at one period the inflammation affects chiefly the peritoneal surface of the uterus, whilst at another it affects its deeper-seated tissues: and in this respect it resembles some other inflammatory diseases of the internal organs, and particularly of the thoracic viscera, which assume an epidemic form.

"It may be observed, from an examination of this abstract, that, in the course of a few days, in the same ward of the hospital, and in patients who were placed in contiguous beds during the prevalence of the epidemic, all the varieties of uterine inflammation which I have described occurred in their most perfect forms. In some, the local and constitutional symptoms were immediately subdued by general and topical bloodletting; but, in other cases, the symptoms were, from the commencement, such as to contra-indicate the use of this remedy, and it was not had recourse to. Such cases usually terminated fatally, in spite of local bleeding and the exhibition of internal remedies; and, on examination after death, the veins, muscular structure, or appendages of the uterus, were found to be the textures most frequently inflamed.

"This fact, that, at different seasons, different textures of the uterine organs are liable to be affected with inflammation, and in varying degrees of intensity, will enable us, in some measure, to reconcile the discordant opinions contained in the works of authors, both with respect to the symptoms of puerperal fever, and the treatment required in different epidemics." (P. 444.)

It is very certain that, until lately, the pathological anatomy of the uterine organs in puerperal women had not received the attention which its importance demanded. Still enough is to be gleaned from the comparatively imperfect records which we have, and to which Dr. Lee refers, to shew that the local and constitutional phenomena of the different epidemic fevers of lying-in women, are to be referred to uterine inflammation.

*Treatment of Uterine Inflammation.* Like inflammation of other organs of the body, that of the uterus varies greatly in severity in different cases, and at different seasons. At some periods, the disease is so mild as to yield readily to anodynes, and to local applications of a soothing nature to the hypogastrium: but where inflammation of the peritoneal covering of the uterus is fully developed, and where the disease prevails in an epidemic form, bloodletting, and the other means for subduing visceral inflammation, must be vigorously employed, or it will, in most cases, proceed to a fatal termination.

"In no inflammatory disease are the good effects of blood-letting more strikingly observed than in the first variety of uterine inflammation, puerperal peritonitis: we do not, however, as Dr. Gordon has stated, possess a remedy in it which will certainly cure the disease in all cases, if early applied. Where the symptoms of peritonitis manifest themselves with great violence, twenty ounces of blood should be immediately drawn from the arm, and in a few hours, if relief is not obtained, sixteen ounces more should be abstracted. The first general bleeding should be followed, without loss of time, by the application of leeches to the abdomen, regulating their number by the severity of the pain and the strength of the pulse. Warm linseed-meal poultices, or fomentations to the hypogastrium, should invariably follow the application of the leeches; and five grains of calomel, with an equal quantity of antimonial powder, should be administered every two or three hours: after the second dose of this medicine, I have frequently exhibited a strong purgative draught, repeating it according to its effect. It will often be found that the pain of the uterus continues with considerable severity after this treatment has been pursued; and that the most decided benefit results from combining half a grain or a grain of opium, or five grains of Dover's powder, with each dose of the calomel and antimony.

"Where the symptoms do not indicate an attack of a formidable nature, we ought not to carry depletion so far: in a large proportion of cases, one bleeding will prove sufficient; and in many the application of leeches alone, with the internal remedies now mentioned, have subdued the disease.

"Oil of turpentine I have seen employed in a few cases, without the slightest advantage.

"Emetics have been administered in puerperal peritonitis, and favorable reports have been published of their effects, both by French and English authors: from the intense pain of the uterus, however, aggravated by the slightest pressure of the hand, or by compression of the abdominal muscles, and from the early occurrence of nausea and vomiting in the worst cases of the disease, emetics obviously appear to be little calculated for the relief of the symptoms. The first favorable report of the effects of emetics was given by M. Doulcet, of Paris, in 1780; and it has been copied by almost all the English writers down to the present period, and has been considered as affording unequivocal proof of the power of these remedies to arrest the disease.

"Doulcet commenced the employment of ipecacuan and kermes mineral in the month of June 1782, according to Alphonse Le Roi, when the epidemic was ceasing: but these means were wholly inefficacious in the months of November and December; for the mortality was greater at this epoch, and at the beginning of the following year, than in 1780, when the remedy of Doulcet was not known; and M. Tenon affirms that the complicated puerperal fever in 1786 was curable by no means then discovered.

"With regard to the treatment of inflammation of the uterine appendages, and of the deeper-seated tissues of the uterus itself, whether of the absorbents, veins, or of the muscular structure, the symptoms, from the commencement, are generally those which contraindicate the use of general bloodletting. In cases where the reaction at the invasion of the disease has been violent, with acute pain of the uterus, and venesection has been employed, the relief obtained has only been temporary, if at all experienced; and in some instances the abstraction of only a few ounces of blood from the arm has produced syncope, or been followed by rapid sinking. Where the local pain is severe, leeches and warm fomentations seem to be the appropriate remedies; but, as far as my own observations go, we are in possession of no remedial means which effectually control those varieties of inflammation of the deeper-seated structures of the uterus, which I have attempted to describe: the French physicians are, however, of a contrary opinion, and are satisfied that we possess a powerful remedy, even in the worst cases, in mercury, employed so as to excite salivation. In one case of uterine phlebitis, I pushed this remedy, by inunction, to a great extent, and brought the system under the influence of mercury in less than twenty-four hours; yet the progress of the symptoms was not arrested, and the patient died, as I had observed others do where the remedy had not been administered. In other cases I have employed mercury to a great extent internally, without the slightest benefit; and it may justly be doubted, from the results of M. Tonnellé's practice, whether or not it possesses the influence he supposes; for of forty-three cases where mercury was used as the chief remedy, only fourteen recovered.

"I cannot conclude this subject, which is unquestionably the most important in obstetrical medicine, without pointing out the necessity which there exists for a full investigation of the means best calculated to prevent the occurrence of uterine inflammation in lying-in hospitals, where its dreadful fatality has been recorded by all writers since the foundation of these institutions: from the registers of the British Lying-in Hospital, Maternité at Paris, the Dublin Lying-in Hospital, and the Tables of M. de Chateau Neuf, it is proved that the average rate of mortality greatly exceeds that of establishments where individuals are attended at their own habitations; and if it should ultimately appear that all precautions are unavailing in diminishing the numbers attacked with the disease, it will then become a subject deserving of serious consideration, whether lying-in hospitals should not be considered, upon the whole, more injurious than beneficial to society." (P. 456.)

*An Account of a Contagious Fever, which occurred amongst the Danish and American Prisoners of War at Chatham, in the Years* 1813, 1814. By Sir Wm. Burnett, knt., m.d., k.c.h., &c.—8vo. pp. 47. Burgess and Hill, London, 1831.

We wish that writers in general would follow the example of Sir William Burnett, and, when they have but little to say, would confine that little to a little space: we should not then have so often to repeat the lamentation of D'Alembert, which has been adopted as the motto of a contemporary reviewer: "L'auteur se tue à allonger, ce que le lecteur se tue à abréger." Single cases and solitary points in practice, which might form respectable communications of half a dozen pages to a Medical Journal, are too often (for such seems now to be the mode,) spun into pamphlets, and pamphlets puff themselves out to the bulk of volumes: indeed, so prevalent has this plan become, and so little scrupulous are bookwrights as to the nature of the makeweight, that the title of a volume is often a very imperfect, nay, rather a most deceitful, guide to its contents: thus, when we take up a work professing to treat of *modern* chemistry, we esteem ourselves fortunate if not more than a tithe be filled by a tedious dissertation on *alchemy,* prefaced with an essay on "the birth, parentage, and education, life, character, and behaviour" of Tubal Cain; or, if we hear a work announced on some particular disease, or even on some ordinary therapeutic means, (as, for example, on dyspepsia, or on diet,) we are agreeably disappointed if not more than half the treatise, or the whole of a first volume, be devoted to the detail of irrelevant physiological researches, or occupied with the anatomy of the digestive organs, too often a mere compilation from the ordinary class books. Had Sir W. Burnett been so inclined, here offered a famous opportunity to fill pages by hundreds, with a preliminary treatise, with notes and annotations on the theory of fever, misstating or refuting the doctrines and opinions of all who have written since the fabled era of Æsculapius, to the not much less speculative epoch of ——, ——, ——; but we erase the names; a general argument is pleasing; we will not personify: it is sometimes as well to write of the living what the proverb entreats us to speak of the dead, "Nil nisi bonum." Sir W. Burnett, however, seems not to be affected with this prevailing *cacoethes scribendi;* and hence we find here, in less than three sheets of far from closely printed demy, all the essential general details of a very malignant fever, which, during the years 1813 and 1814, raged in the prison and hospital ships stationed in the Medway and off Sheerness.

This memoir, although, like the poet's freedom, "longo post tempore venit," is really an interesting paper, and affords another proof, if indeed another proof were wanting, of the spontaneous origin of fevers, "born in a garret, and in a kitchen bred," as well as of fevers excited by local causes becoming, in their course, not only infectious, (limiting that term to its most appropriate signification,) but also contagious in the highest degree; being conveyed by persons sick from the original source to previously healthy situations, and by them communicated to previously healthy people; and thus exemplifying the truth of the author's text, 'Melius est cavere semper quam pati semel."

The fever in question Sir William declares it to be his "object to prove was generated from local and adventitious causes in one of the ships, from whence it was communicated to the other ships in succession, and eventually diffused through the whole of them, unquestionably by contagious agency;" and we fully agree that

"The uninterrupted chain of evidence which the history of these fevers will be found to present, places their essentially contagious property in a conspicuous light, and will probably be considered to be more than sufficient to remove from every unprejudiced mind all reasonable grounds for disbelief of their infectious origin and nature." (P. 3.)

Early in the month of February 1814, a report was made to the Transport Board, by the late Mr. Thompson, surgeon of the Bahama prison ship, at Chatham, that a fever, which for some weeks had occasionally shewn itself in that ship, had considerably increased, there being eleven Danes on the list with the disease, and several convalescents. The number of sick was, by the 8th February, increased to twenty-one; and on the 10th, in the Bahama alone, they amounted to fifty. This fever likewise shewed itself on board several other ships, as the Defiance, the Kron Princen, and the Fyen; for

"The Fyen, having a large sick berth, was appropriated, agreeably to the directions of the Board, to the reception of sick men from other ships: amongst these were several cases from the Bahama; and Mr. Towns, the surgeon, states in his journal, that 'the Fyen, previous to receiving the sick Danes from the Bahama, was very healthy; but notwithstanding every precaution was taken to prevent any communication between the hospital and the prisons, typhus made its appearance shortly afterwards amongst the prisoners, marines, and women.' The fever appears to have extended to nearly sixty persons on board the Fyen, and in every respect bore a strict resemblance to the worst cases of the Bahama." (P. 7.)

Seventy-eight Danes were removed from the Bahama to the hospital ship, on the 12th of February; and on the following day, ten were sent from the Defiance; by the 6th of March, forty-three additional cases had been received; and out of the 157 then sick, forty-five died, twenty-four previous, and twenty-one subsequent, to the date now mentioned: at this time

"The hospital contained 126 patients, twenty of whom were reported to me to be 'dangerously ill, thirty-six very ill;' many of them were covered with petechiæ and vibices, and in several the feet and legs were gangrenous, the disease still extending.

"The Bahama and Defiance were in an equally unfavorable state, as the fever was making rapid advances in each of these ships; added to which, the surgeon who had been appointed to succeed Mr. Thomson (which latter gentleman fell an early victim to the fever,) was in so ill a state of health as to be unable to perform his duty; and Mr. Johnson, the surgeon of the Defiance, was at this time confined to bed, labouring under a very severe attack of the prevailing disease, and was also succeeded by another surgeon. From the Bahama, therefore, and Defiance, I could get no satisfactory account of the progress of the disease, nor, in the former, of the number still sick on board; but I found a large sick berth full of patients labouring under fever, most of them very dangerously ill. In the Defiance I found sixty-three cases, fifty-five of which were in bed, and the greater part of them exceedingly ill." (P. 9.)

As an evidence of the contagiousness and malignancy of this fever, we may notice that

"During the progress of the disease, every medical officer employed (except, I believe, one assistant surgeon and myself,) suffered an attack of the fever, and one died. Many of the ship's company and marines of the sickly ships were at different times attacked; but these being sent to the Sussex hospital-ship at Sheerness, I had not an opportunity of personally ascertaining the termination of their cases; though, I believe, the deaths were not numerous.

"The total number of prisoners of war taken ill after the 6th of March, was 518; of these, sixty-one died." (P. 13.)

The symptoms of the disease appear to have been very various, especially in the incipient stages, but still, on the whole, they do not seem to have differed essentially from the ordinarily varied modifications of typhus; and when the fever became confirmed, "the range of symptoms was more common to the whole."

Sir William mentions one interesting pathological fact, which, indeed, others have also occasionally noted, viz. that "in the course of the disease several evacuated lumbrici, and in one case I observed a large portion of tænia discharged: in fact, the condition of fever is generally observed to be incompatible with the existence of intestinal worms: they are commonly discharged dead before the termination of the disease." (P. 16.)

In the account of the morbid appearances and treatment, we do not find much to arrest our attention, or to excite our comments: occasional and guarded bloodletting, a moderate exhibition of mercury, purgatives, and the ordinary febrifuges, with the exception of emetics, seem to have been the most beneficial: against the latter, our author enters a vigorous protest.

"In several instances emetics had been administered; but I do not know one case in which they were attended with the smallest advantage. I am aware that, in stating the foregoing opinion on this subject, I shall offend against the laws of orthodoxy; but I feel bound to declare (after as extensive a practice in contagious fever as has, perhaps, fallen to the lot of any one,) that I never, on any occasion, when treating this disease, derived benefit from the use of emetics; but, on the contrary, during the period the hospital for prisoners of war at Portsmouth was under my direction, where I have often received twenty and thirty men in a day, and, indeed, sometimes very many more, labouring under contagious fever, I uniformly found that, whenever a patient had taken an emetic previously to his coming to the hospital, the disease was much more protracted, infinitely more difficult of cure, and consequently, in general, far more fatal." (P. 26.)

The causes of this fever seem to have been traced with much care and very considerable success; for the more minute details, we must refer to the essay itself: the conclusions which the author draws therefrom, and in which we think, from the premises, he is fully justified in doing, are all for which we can afford room. Having premised that the illness of Mr. Johnstone, surgeon of the Defiance, of Mr. Scott, surgeon of the Bahama, and the death of his predecessor, Mr. Thomson, (the two former of whom suffered severely by, and the latter fell a victim to, this fever,) rendered the task more difficult, he observes,

"Notwithstanding the foregoing difficulties, I trust I have been able to trace the origin and progress of the disease, in such a way as to leave no doubt respecting either.

"It results, then, that occasional cases of fever began to make their appearance in the Bahama in the early part of January, at which time they were viewed without any kind of alarm by the surgeon, and it is certain that at this period the fever had made very little progress; for we find that, out of 324 prisoners sent from the Bahama to the Defiance, on the 14th of that month, only two cases occurred in the space of a fortnight, viz. one on the 25th, and the second on the 30th of the same month; and even in the Bahama herself, the *fons et origo* of the disease, there were only five cases on the list on the 6th of February.

"I have before mentioned the inclement state of the weather, in consequence of which the prisoners took every method in their power to exclude the external air: they had, moreover, a great quantity of dirty clothes, and were exceedingly indolent; so that it was with the utmost difficulty any thing approaching to a clean or well-ventilated state of the prison decks could be obtained, though every means were used for that purpose; and it was necessary to employ some of the French prisoners, (who for the usual pay of the *employé* undertook this office,) that even this might be done. When these things, together with the depressed state of mind incident to such a situation, are taken into consideration, there seems just reason to conclude that the fever in question originated amongst themselves, from animal effluvia; became concentrated, from the total want of ventilation; and that it afterwards assumed an infectious form, and propagated itself whenever there was sufficient communication with those infected.

"There is, therefore, no difficulty in accounting for the appearance of fever in the Fyen; which, indeed, Mr. Towns, the surgeon, traces distinctly to the reception of patients labouring under that disease from the Bahama; and there can, I think, be no reason to doubt that the two men, named Hughes and Gilive, introduced the disease into the Kron Princen: but it may also be added, that all these ships having been solely appropriated to the reception of Danish and American prisoners, there were, no doubt, instances of communication and intercourse, which it would be impossible to trace.

"I have already mentioned that Mr. Thomson, surgeon of the Bahama, fell an early victim to the fever. Mr. Scott, the surgeon who succeeded him, was soon also attacked; as were Mr. Brennan, the surgeon who succeeded Mr. Scott, two English and two French assistants employed on board. In this ship, the ship's company and marines suffered greatly, there being no less than forty-one attacked, viz. two English assistant surgeons; one lent from the Cumberland seventy-four, who was taken ill a few days after he came on board; and two French assistants; seven of the ship's company, and twenty-nine marines, being nearly the whole party of the latter corps embarked: moreover, several of their wives were also taken ill with the same fever.

"The attack of the marines succeeded each other so quickly, that for a short time the cause appeared to be something more than their usual duty at the hatchways, or stairs leading to the prison decks, could account for: it was, however, at last easily explained.

"The place in which the marines lived and slept, had loopholes for musquetry opening upon the main prison deck, by which means the infectious effluvia readily found their way thither: after these loopholes were boarded up, the disease in a great measure ceased amongst them; thus affording the strongest proof of the deleterious effects of the effluvia, and of the efficacy of separation.

"In the Defiance, the fever extended to the surgeon, six marines, and three seamen. In the Belliqueux, amongst the prisoners of which ship the disease had nearly ceased before their removal from the Defiance, two of the marines were attacked; and these were fresh men from the shore.

"Under such circumstances, it will not be a matter of surprise that the medical officers and attendants in the Trusty hospital ship did not escape: accordingly I have to mention, that my friend Dr. (then Mr.) Dobson had a very severe attack, and also several of his assistants. Seven of the nurses, also, were infected, and two of the latter died.

"I think the foregoing detail will be enough to satisfy the minds of the most incredulous, that the fever in question was undoubtedly of an infectious nature; and were I to have recourse to the published works of others, it would be a matter of the greatest facility to adduce many parallel instances in such diseases. I shall however (though even that is almost a work of supererogation) content myself with giving a few extracts from the medical journals of the surgeons of the fleet, which will, I think, fully corroborate the views I have taken on this occasion." (P. 35.)

The instances here adverted to, occurred in his Majesty's ship Elizabeth, the Dolphin troop ship, and his Majesty's ship Cambrian, and are well worthy perusal: from the first-named vessel, seventeen cases were sent to Haslar hospital, on the 28th December; eighty-one on the 29th; twenty-four on the 2d January; forty-one on the 3d; fifteen on the 4th; five on the 5th; nine on the 6th; nine on the 9th; twenty-eight on the 10th; four on the 11th; and fifty between the last-named day and the 17th; after which, ten other cases only occurred: besides which, the captain's clerk, having visited a messmate at the hospital, was taken ill on the 22d, and died on board on the 31st.

In the second vessel, viz. the Dolphin, to such a height did it (fever of a typhoid and infectious nature,) "attain, that at one time there were no less than fifty-six of the ship's company, and 176 prisoners, ill with this fever. The total number of prisoners attacked is not stated; but so prevalent was the fever, that "it extended to 130 of the ship's company." Furthermore,

"The Dolphin having been ordered into Portsmouth harbour to refit, in the weakened state of the crew from disease, a party of men were ordered from the St. George, then at Spithead, to assist in transporting her thither. This took place on the 3d of January: on the 10th of that month, fourteen cases of fever were sent to the hospital from the St. George; and from this time to the 19th, many cases were sent daily, amounting to 133; three were sent on the 21st, after which the disease appears to have ceased." (P. 42.)

The case of the Cambrian differs not materially in its purport from the preceding; and, as the cumulative argument seems to us needless here, we shall not make further extracts, but let our author take his leave of our readers in his own words:

"In conclusion, I beg to remark, that these latter statements have been extracted from authentic documents, to which my official situation has afforded me access; and the events described having occurred in situations remote from each other, at various periods, and under the observation of different individuals, who agree in all the essential points I have aimed at establishing, it will probably be admitted that they constitute a body of incontrovertible evidence, in support of the position that fever, of an actively contagious nature, is commonly generated whenever masses of men are confined in a limited space, under the insalubrious circumstances of crowding, want of cleanliness, and a vitiated atmosphere; more especially when aided by the influence of the depressing passions." (P. 46.)

---

*First Principles of Medicine.* By ARCHIBALD BILLING, M.D., Fellow of the Royal College of Physicians, &c.—8vo. pp. 131. Underwood, London.

THIS little volume, which, were it not for the boards, we might almost esteem a pamphlet, contains in its 130 more matter truly valuable than we often find spread through thrice as many pages: not that it abounds in facts or theories absolutely original, but that, in wending through the hosts of too-often irrelative experiments, and the bewildering mazes of speculation, the author displays sound judgment and great power of discrimination; estimating each according to its worth, and associating with much skill those which throw the greatest light upon the important topic he has professed to treat, so that such discoveries and doctrines as really belong to others, by rightly adapting, he has almost made his own.

Persons who confine not their studies to the works of one sect or language, but retrospect and circumspect the physical sciences, medicine more especially, cannot fail to have been astonished at the very contradictory views and modes of practice which, at different times, and among different people, have been published, praised, and pursued; and still more must they have been astonished at the comparative similarity of effect resulting from such different treatment; e. g. to take one of the latest instances, and which even now is present, the great difference we read of in the treatment of Indian cholera, and the little difference proved to be found in their relative success. This probably arises, not only in the instance given, but in many others, as in fevers, inflammations, &c., from not duly estimating the differences of attendant or secondary circumstances in the disease, and attendant or secondary effects of the remedial means; so that the treatment of each sect which is most successful in the types which have formed their several models, becomes less so in those which are the models of their opponents. Thus it is that the balance of success is struck, and thus is it that, although science advances, it advances (if we may so express ourselves,) in different directions, and, although knowledge is much increased, its practical application is not made, to the utmost, available for the public good. What work, then, can be more useful than to reconcile the apparent discrepancies of contending theories, and to associate the practical advantages of the several schools into a common fund, or army, for future profit, or for future conquest.

This project Dr. Billing has, in some measure, very unostentatiously attempted to achieve in his "First Principles of Medicine," with what effect we shall leave the following extracts to determine. The sketch here given, of course, is very incomplete, yet, so far as it goes, we think it in general good: the views are liberal, and becoming a medical philosopher. We shall look with anxiety for a more extended essay, of which the present can only be esteemed the harbinger.

Reasoning in medicine is, of necessity, for the most part, reasoning by analogy: indirect experience and direct experiments are the premises whence conclusions must be drawn: hence, when (as, in the present state of science, too often is the case,) these are wanting, practice must become, as in fact it is, empirical; but then even such empiricism, in the hands of the philosopher, will furnish the experience and experiments from which sound conclusions may be deduced; so that empirical practice affords those very premises for reasoning whereby empiricism itself is regulated and controlled. Thus theory and practice tend reciprocally to advance each other, and mutually improving and being improved; for, as our practice improves our theory, so does our theory improve our practice; and we therefore highly approve of the summary sketches of modern physiology blended with pathology, as the rationale of medical practice met with in this work. We cannot pretend to abridge a treatise, in its construction almost aphoristical, so as to form a continuous essay; there will, of necessity, be frequent chasms between our extracts: to fill the void, we must refer to the mine whence they are drawn, rather as samples of what the industrious workman may there expect to meet with, than as proofs that the rich vein thus opened has been exhausted. Indeed, we can scarcely regard our author as more than a pioneer, who has somewhat cleared the way and sunk a shaft; or, at most, as having reduced some specimens of the ore, to shew what future metallurgists may be encouraged to expect.

In the very beginning of this volume, we find an important case entered, to which *morbid* anatomists, if they desire the adjective to apply not to the examiners, but to the things examined, would do well especially to attend.

"An accurate knowledge of the functions in their healthy state is the more necessary, because considerable deviations from the ordinary routine take place without disease, and as they are frequently much disturbed without any discoverable alteration in the structure of the organs having taken place, morbid anatomy alone will not be sufficient to elucidate all causes of disease; whilst, on the other hand, it is necessary to be aware that a considerably diseased change of structure may exist, with little or no interruption of function." (P. 1.)

Perhaps the author's physiological views and illustrations may by some be thought rather too purely chemical and mechanical: they are, however, often quaint and always ingenious; e. g.

"During health, the process of nutrition is thus carried on: the food swallowed is digested by the action of the gastric juice in the stomach, that is, it is converted into a grey pulpy mass called chyme, which passes on into the intestines, where it is mixed with the bile. The use of the bile is to unite with and separate the feculent parts, as white of egg is used to clear wine. Now, if a pulpy mass be allowed to stand in a vessel, the solid parts will settle to the bottom, but if rolled about in the hands, or in the manner effected by the peristaltic motion of the intestines, the more solid parts are kept in the middle, whilst the surface of the mass is the moistest; and thus a whitish liquid called chyle, which was disengaged when the bile united with the feculent matter, and which caused the chyme to appear grey and constitutes the new nourishment, is kept next the coats of the intestines, where it is sucked up by the tubes called absorbent vessels; and these absorbents, on account of the white chyle seen through them, are called lacteal, (milky.)" (P. 3.)

And again:

"The body is nourished by the arteries depositing, in appropriate parts, the various constituents of the blood, which are sent through them by the heart. In this way muscles, bones, membranes, &c. grow and are nourished, for the blood contains the constituents of each; fibrine, &c., for instance, to make muscles; lime, &c. for the bones; albuminous and watery fluid for the formation of membranes, and to supply the secretions and exhalations, which are necessary to lubricate the mucous and serous membranes.

"Though a consideration of the phenomena resulting from these depositions will assist us in our explanation of disease, we cannot exactly ascertain how the depositions themselves are originated. Do arteries build up a bone merely by the addition of homogenous matter? and, are the secretions and exhalations modified by the caliber of the minute branches, admitting only the vapoury parts to the surface of the serous membranes and of the skin, whilst they permit the transparent fluid parts of the blood to pass to the mucous surfaces, and keep back the red globules. This mechanical explanation might suffice, in part, in the instances adduced; but when we come to the nutrition and renewal of muscle, and the formation of peculiar secretions, we must look for some still uncomprehended agency, which modifies the materials conveyed by the arteries, whilst they are depositing; even with respect to the deposition of bone, this agency is required to solidify the new particles, which are fluid in the blood: this power can be no other than chemical; the processes, when examined, will be found to be chemical precipitations by which new matter is deposited, and decomposition by which old matter is separated, and then carried off by absorbents, and thus the support of the frame in health and the changes of disease proceed. In this investigation we may advance a considerable way, though we cannot come to the knowledge of the ultimate principle on which organic life depends, or we should be able to construct a man: as an instance how far we can go, we can analyse bone, and we may explain how bony matter is deposited from the blood by precipitation, and we know that the shape depends on the periosteum, or membranous mould in which it is cast: but here we stop; we cannot discover how, in the minute embryo in the womb, the membranes were first determined in their shapes; we here arrive at the confines of our knowledge, and must confess an infinitely wise First Cause, who does not permit us to know more than the phenomena by which we can judge how, in many instances, to avail ourselves of the means to regulate the complicated apparatus which he has endowed with life.

"The deposition of bone is a combination of chemical precipitation and crystallization, modified by vital actions; as, for instance, when there is periosteal membrane, we see that it keeps up a vital state of bone, whether in the bone of a leg or a tooth; when there is no membrane attached, as in the enamel of the tooth, crystallization with the temporary membrane which forms the mould decides the form of aggregation; in case of fracture of a bone, the surrounding parts decide the form of the callus which reunites it. Whilst bone is growing, (as shewn by the common experiment of feeding young animals with madder so as to produce variegated deposits) there is a change as to disposition of the bony matter going on, but there is no reason to suppose that the substance of a healthy sound bone of an adult is changing, no more than a tooth, or the wall of a castle, though there are preparations ready to repair a breach, if made.

"The arteries of the periosteum are always ready to repair, and as soon as they get notice they begin to deposit (the bricks and mortar): What is the notice? Why do they deposit when they get it, more than they did before? The notice is inflammation, whether it be from a fracture, a blow, or other accident, and the vessels by this inflammation becoming distended, (with or without rupture and extravasation,) and the part spongy, there is, if not stagnation, a sufficient retardation of the blood to allow of crystallization of bony matter. When do the workmen cease depositing? When the spaces prepared by the inflammation are filled; if there be not enough of bone deposited to unite a broken limb, the surgeon often rubs the broken ends against each other to excite new inflammation, because, strange as it may sound to the unlearned, we cure in many instances by producing inflammation. We may thus account for the hopeless work of the vessels in the toothach, the injury is in a part which cannot be repaired, as being destitute of membrane; hence the inflammation excited in the sound part produces only a useless effort, as we see sometimes evinced by morbid growth at the point of the root, but no repair of the mischief, so that the tooth must be removed altogether. The same effort which repairs, if excited morbidly, produces diseased growths, such as bony tumors from syphilitic or other inflammation of the membranes of the bones." (P. 4.)

In the following quotation, the actions of the heart and arteries will be found well and clearly stated.

"The heart contracts by muscular action and relaxes alternately, the arteries keep up a constant contractile pressure on their contents, not an alternate contraction and relaxation, but a continued contractile effort, which is overcome by the action of the heart; when there is much blood sent into them, they are distended; and if there be little blood sent into them, as after hemorrhage, their tendency to contract causes them to close, so as to keep always full, and to preserve a constant stream of the blood, even during the temporary relaxation of the heart; and the arteries yielding and adapting themselves to the pressure of the heart, and recontracting on their contents, whilst the heart is relaxed and filling, is the cause of the equability of the stream in the veins, nay, the stream even in the arteries is much less in jets than is supposed by those who judge from the mode of its flowing from a wounded artery; for though, when there is a free escape from the wound, the impulse of the heart causes an unequal stream, it must be remembered that in the tube, unwounded, that force would have been expended in stretching the artery; whereas the artery, when wounded, ceases to be other than as a simple tube, the elasticity not being called into operation, on account of the escape of the blood from the wound.

"The most simple mechanical illustration, perhaps, is the double bellows of a smith's forge, which keeps up a constant current of air, though the handle works with intermissions, so that the blast into the fire would be in puffs, if it were not for the weight of the upper half of the bellows, which keeps forcing out the air in a continued current, whilst the hand is drawing back to make another impulse.

"It has been supposed that the circular fibres of the arteries were muscular, and that they contracted and relaxed at each pulse, and that the throb felt was caused by a dilatation of the artery; those fibres are not muscular, but more approaching to a ligamentous tissue, firm and (though elastic) not yielding to the force of the heart, but, on the contrary, preserving the caliber of the artery uniform, as may be seen by laying bare an artery in a living animal, or when the artery is laid bare in an operation; it is longitudinally that the arteries are stretched at each injection from the heart, by which their capacity is increased; the consequence of which, from their being bound down in various places, is, that there is a serpentine motion in the artery where it is at all loose.

"The fibres of the middle coat of the artery being arranged circularly, allow of the separation laterally, and thus accommodate themselves to the elongation of the tube, whilst they resist its dilatation. Now it may be thought that the movements of arteries, seen at the wrist and in the temples, is their dilatation; but it is the serpentine motion caused by the alteration of the curve, the artery being elongated at each injection from the heart.

"Where the artery is perfectly straight, you may lay it bare and scarcely see it move, but the moment you compress it with the finger, or tie a ligature around it, you perceive it pushed at every pulse. To illustrate the deception of the sensation which the pulse gives, as if the artery were dilated at each beat: if a long vein removed from the body have a syringe adapted to one end, the other being raised, or arranged with a spring valve, which yields to the jets so as to keep it full, and fluid be sent through in jets, it will, upon pressure by the finger, give the sensation of dilatation, but the eye perceives none: again, if any one grasp the leathern tube of a fire or garden engine, the sensation given, will be that of its expanding in the hand at each stroke of the pump, but the eye contradicts the sensation: it is merely the tendency to resume the cylindrical form from the outward pressure of the fluid, but not expansion." (P. 9.)

We doubt whether all our modern physiologists will agree with Dr. Billing as to the cause of the return of the blood to the heart through the veins, and the distention of the right auricle: the passage is too long for extraction; suffice it, to state his opinion negatively, that it is

"Not any suction (to use a vulgar expression) of the heart, or suction of the chest, as has been attempted to be proved; no effect of vacuum and atmospheric pressure; there is no suction (no atmospheric pressure) during natural respiration, for the glottis is sufficient to admit a free current of air; it is only in croup or laboured respiration of any kind that there can be any effect of atmospheric pressure." (P. 12.)

We think the learned Doctor less happy than usual in his physiology of the generation of animal heat, and in its illustrations: he says,

"The animal heat has been accounted for in different ways by several ingenious physiologists: from the aggregate of their opinions and experiments I deduce, that heat is extricated all over the frame, in the capillaries, by the action of the nerves, during the change of the blood, from scarlet arterial to purple venous; and also whilst it is changing in the lungs from purple to scarlet.

"There is a perpetual deposition, by the capillary system, of new matter and decomposition of the old all over the frame, influenced by the nerves; in this decomposition there is a continual disengagement of carbon, which mixes with the blood returning to the heart, at the time it changes from scarlet to purple; this decomposition being effected by the electric agency of the nerves, produces constant extrication of caloric; again, in the lungs that carbon is thrown off and united with oxygen, during which caloric is again set free; so that we have in the lungs a charcoal fire constantly burning, and in the other parts a wood fire, the one producing carbonic acid gas, the other carbon; the food supplying through the circulation the vegetable (or what answers the same end, animal) fuel, from which the charcoal is prepared which is burned in the lungs." (P. 18.)

There are certainly many points of similitude between the nervous and electric fluids: to them we have so frequently adverted in our Journal, that they do not now need repetition; we therefore pass over our author's conspectus of what at present must be considered merely hypothesis, as to the cause of nervous energy, to what is more certain knowledge, viz. its effects; for upon this point we find some very apposite observations.

"Whatever nervous influence may be, or however generated, we know that the energy of parts depends upon a something that is communicated to them by the nerves in conjunction with the brain and spinal marrow; that while parts are supplied with this nervous influence, they retain their power of action, and not longer; arteries become insusceptible of impression from external agents when the nervous energies are low; when the vital powers are sunk, the capillary arteries cease to secrete; various phenomena of inflammation are the effects of healthy action of the heart and arteries; and we find, when nervous energy is deficient, that parts which had advanced to a certain stage of healing become flabby, as in stumps after operation when the patient sinks; also when the powers of the constitution, the nervous energy fails, the application of boiling water will not cause any effusion of serum under the cuticle (called blistering), nitrate of silver will have no effect upon ulcers except chemical decomposition, not that astringent effect which is the result of contractility depending on vitality. It is well known likewise that a blister not rising from a cantharides plaster is a bad sign, but no vesication will take place even from boiling water, when the vital powers are sunk, as the heart has not power to effuse serum: this is a more satisfactory example than cantharides, because the effect of the hot water goes so far as to produce the local injury, for the cuticle may be separated or loosened by the mere chemical effect of the heat; but this takes place equally in a dead body: it is the extravasation of serum under the cuticle to raise it up which requires vital action." (P. 19.)

The following we think an extremely good summary of the facts arrived at in the investigation of a problem, that has been the subject of much dispute: it is also still further interesting, as it includes the rationale of a very important improvement in modern practice, viz. the use of a very strong ointment of lunar caustic in ophthalmia. This is a mode of treatment so incalculably superior to the not yet exploded system of bloodletting, that it is making rapid progress; but still, from what we have heard and seen, it is often adopted without the philosophy, beautiful as it is, being clearly understood.

"With respect to the action of the heart, all are agreed that its action is contraction, by which the blood is sent forward in the arteries, and that the power of the heart's action is measured by the pulse, when there is no organic alteration, such as ossification of the valves at the beginning of the aorta, aneurism, &c.

"The action of the arteries also is acknowledged to be contraction, whether muscular or not, but there is some difference of opinion as to the state of the arteries in inflamed parts. It is very common to say, that in an inflamed part there is an increase of arterial action; but a consideration of the phenomena, and of the nature of arterial action, will shew that in inflamed parts the capillary arteries are weaker in their action, that there is diminished arterial action, for the action of arteries is contraction; now the arteries in inflamed parts are evidently larger than before, less contracted, that is, acting less.

"An inflamed part is redder and swelled; where the vessels are visible, as in the eye, we can see the redness is caused by the minute vessels becoming larger, so as to admit red globules of the blood, which before admitted only the more fluid and transparent part, or the minute vessels carrying red globules becoming larger; this enlargement of vessels is not from increased action, but, on the contrary, from their action being diminished, their giving way, and being dilated by the injecting force of the heart. The way to diminish the inflammation is by increasing the action of the arteries, as by cold or astringents, which make the arteries contract, that is, increases their action; so that so far from the arteries in an inflamed part being in a state of increased action, one of the means of diminishing inflammation is by increasing arterial action in the part inflamed. It is common to remark the throbbing of the carotid arteries as increased action, but the more they throb it shews that they the more yield to the injecting force of the heart; when the eye, or any other part, is injured by heat, or a stream of cold air, a blow, or cantharides plaster applied to the skin, &c., the part becomes redder from the vessels enlarging, and carrying either more red blood, or admitting red blood where there was none before. Now in this first and simplest instance of inflammation, the heart does not act more strongly than ordinary, not affecting the pulse; so that the capillary arteries evince debility, having given way, when there is no more force than they bore before without distention: from this they sometimes recover of themselves, gradually contracting to their natural size; or if not, the simple application of cold, or astringent lotion, makes them contract, and the redness disappears.

"On the other hand, by savine or cantharides ointment we can produce an inflammation, such a relaxation of the capillaries that those which can be dilated, are, and separate from those which are confined by firm surrounding substance, by which means warts are thrown off, which has been called increasing vascular action beyond what they could bear; it is increasing the size of the vessels, but not their action. It is the opinion of some persons even at the present day, that the motion of the blood is accelerated in inflamed parts, though the experiments of Parry and others prove the contrary to be the case, as follows from the capillaries being enlarged, inasmuch as when fluid passes through a given space, the current beyond that will be slower in proportion to the wideness of the channel, as every one must have observed in a wide part of a river, where the current becomes slower; and the same may be observed by passing water mixed with grains of amber through a glass tube with a bulbous enlargement in the middle, the current will slacken in the bulb and resume its velocity beyond it.

"Some will allow that the capillary arteries, where the blush of inflammation is, are weak, as they visibly have given way, bnt they still talk of increased arterial action, and say that the arteries around or leading to the inflamed part are in increased activity, as a part of the condition or what keeps up the inflammation; not considering that an increase in their action would be contraction, and so a diminution of the flow of blood to the inflamed part; wherefore an increased action in the arteries both in and leading to the inflamed part is just what is required to diminish the inflammation.

"The more the heart acts, the more of course it forces the arteries of the inflamed part, and the pulse shewing the degree of power of action of the heart is erroneously by some considered as an evidence of arterial action, as the throbbing of the carotid arteries, for instance; the heart then acting against the capillaries, if we cannot get them to act strongly enough to resist its force, we are obliged to diminish the force of the circulation, either by taking away blood, which diminishes both the quantity of blood sent to the arteries and the action of the heart itself, and in this way we leave less for the arteries of the inflamed part to do; or, we can lower the force of the heart by medicines, such as digitalis, &c.; here, for illustration, the simplest cases of inflammation have been taken, where the heart is acting naturally, the inflammation being from injury." (P. 20.)

We cannot forbear adding the following to our extracts.

"The difference between congestion and inflammation is that in congestion there is mere distention of vessels, in inflammation there is more or less alteration of tissue, connected generally with deposition in some way of coagulable lymph; the moment congestion is relieved the parts are in their natural state, but even after inflamed vessels are unloaded there is time required for recovering their natural state; a good example is the congestion of the lungs in fevers, which often leaves no symptom when the fever is relieved; but after inflammation of the lungs has been stopped, they require time to regain their natural state." (P. 24.)

It is always well to have a clear and decided meaning to the terms which we employ, and, although it may be often inconvenient to change the language of the profession, which has become the adopted language of the public, still, when a better physiology teaches us better things, we must at any rate ingraft better meanings on our old, if we cannot expunge them and introduce better terms. This our author attempts to do, and from several examples we select the following.

"It is sometimes remarked that local irritation detains the blood in a part, which may be explained by the increased capacity of the vessels causing a slower current, as before stated; besides this expression, and the terms congestion and inflammation, there is another word, determination, used to express an habitual reception of more blood than natural in a part, as 'determination of blood to the head with throbbing of the carotids,' the throbbing of the carotids has been already explained not to be active but passive:

now the word determination, in ordinary language, implies that blood is sent there in particular, but the heart has no power to direct any blood to one part more than another, although if in any part there is an unusual relaxation of the vessels, they will receive more than usual; as, when the water is sent through the great pipe of one of the water works it cannot be determined to any house in particular, but whichever house has the largest cistern will receive most water: by this I wish merely to illustrate, that what is called determination is not active but passive; the term also used by Bichat, of the blood being drawn or invited into an inflamed part, may be explained on the same principles." (P. 27.)

The observations on secretion and excretion, the growth and disappearances of tumors, &c. are well worth perusal: we only regret that space forbids us to do more than refer to them, because we must reserve the little that remains for another purpose.

"It is necessary here to mention the distinction between sedatives, stimulants, tonics, and narcotics, a great confusion of language and ideas having prevailed on these subjects, as for instance, any medicine which made a person better without evident effect on the bowels, kidneys, &c. was called a tonic, and insomuch as it restored tone to the system undoubtedly had a tonic effect; now this is the case so often with wine in debilitated habits that it is no wonder stimulants and tonics became almost synonymous, and the common mode formerly of administering bark in port wine increased the error, and bark was thought stimulant; we have a difficulty too in distinguishing qualities of medicines from many of them having two principles combined, as will be pointed out directly: we may however get very nearly pure examples of each, stimulant, sedative, narcotic, and tonic.

"A stimulant is that which through the medium of the nervous system increases the action of the heart and other organs, by calling forth the nervous influence or by facilitating the extrication of it in them; for example, wine, brandy, and other spirits, the product of fermentation.

"A sedative is that which diminishes the action of the heart and other organs by repressing the nervous influence, for example, digitalis, and green tea, which, though called a stimulant by some, was shewn by Dr. E. Perceval to have an effect similar with digitalis: green tea in excess produces a sense of anxiety and oppression of the chest, with intermitting weak pulse, nausea, &c. I acknowledge that the new Italian doctrine of medicine has given us more definite ideas on this subject, and the practice of that school elucidated the properties of remedies; they use the word stimulant as we do, but they have discarded the word sedative on account of its being confounded with narcotic, &c. I think I have drawn the distinction clearly without adopting their new term of contra-stimulant, which seems unnecessary, if sedative be separated from narcotic: their term contrastimulant, however, is good, as it can never be mistaken.

"It has been often asserted that there is no such thing as a direct sedative or allayer of action, but that the sedative effect was only the result of exhaustion from stimulus, arguing from the stupid state which comes on in intoxication from fermented liquors, and from opium, which is observed to be stimulant in small quantities, before enough is taken to produce stupor. In the first instance, the brandy produces stupor in reality by excess of stimulus exhausting nervous influence and perception of the brain, as looking at the sun will take away the power of the optic nerves by excess of stimulus, and too great noise will cause temporary deafness.

"The case is different with opium, for opium contains two principles, the stimulant and the narcotic: this is not now matter of speculation, because they have been separated chemically, and the narcotic part can be used to produce sleep, without the stimulant. The stupor from opium was said to be the sedative effect subsequent to, or produced by, the exhaustion of the stimulus; but this is not the case, for the stimulant part being taken away, the narcotic part produces the sleep just as certainly." (P. 44.)

"Tonics are those substances neither immediately calling forth nor repressing actions, but giving power to the nervous system to generate or secrete the nervous influence; in other words, making the nervous system stronger, by which the powers of the whole frame are increased, but doing so without any evident phenomena of immediate excitement, as by stimulants, or of repression, as by sedatives." (P. 51.)

So many speculations have been adventured as to the *modus curandi* of mercury, that it would not be fair to withhold our author's doctrine.

"The term tonic is applicable also to all those medicines which cure inflammation without being either stimulant, or directly sedative or depletory, commonly called antiphlogistic, and this will lead us to the rationale of tonics on the nervous system; there are various cases in which bleeding, cathartics, emetics, and other antiphlogistic remedies have not power to stop inflammation, and are yet wearing down the constitution: under these circumstances the great resource has been mercury; there are cases also in which arsenic, bark, opium, or other medicines are more applicable, but the great nostrum has been mercury, and yet, though so useful in the most ignorant hands, it is difficult to account for or denominate its action; it is often called a stimulant, and yet it cures inflammation when all stimulants are carefully withheld, and so coincides in its action with the sedatives, and might as justly be called a sedative, but it also cures inflammation in debilitated habits, when wine and other stimulants are obliged to be given; I therefore consider it neither stimulant nor sedative, but tonic, that is by the specific action on the capillaries, whether directly on their tissue or through the medium of their nerves, it causes them to contract, when (though all the injecting force of the heart were taken off by sedative treatment) they would not have had power to close; when introduced into the circulation, arriving at the capillaries, which cannot be otherwise come at, giving them tone to contract analogous to the effect of an astringent applied to external sores. Liquor arsenicalis, nitrate of silver, sulphate of copper, colchicum, &c., have a similar action, some more applicable than others to particular cases. This, I say, is the rationale also of what is called stimulating the secretions of internal organs: when their capillaries are weak, they have the tone restored by mercury, and the secretions thus renewed; but it should not be forgotten that mercury, like some other tonics in excess, becomes poisonous, and may cause inflammation in other parts, as it does in the gums, on the principle adduced before, that one degree of contraction of the capillaries is necessary to reduce inflammation, a still further degree will stop nutrition, and bring on wasting and disease, as syphilis is starved out sometimes at the expense of the constitution." (P. 55.)

The distinctions laid down between narcotics, stimulants, and sedatives, will be found of much practical utility to students.

So many and so just are the practical remarks, rendered still more impressive by the terseness of the language in which they are couched, occurring in the latter, or more especially therapeutical part of the volume, that it would be difficult to select any one or two as samples of others, which are directed to different objects: we therefore, still regretting our unwilling neglect of fevers, idiopathic, symptomatic, and eruptive, tetanus, dropsy, irritation, &c., which would encroach too largely on our pages, are compelled to close our notice of Dr. Billing's useful and instructive little work.

